# Exploiting the Zonulin Mouse Model to Establish the Role of Primary Impaired Gut Barrier Function on Microbiota Composition and Immune Profiles

**DOI:** 10.3389/fimmu.2019.02233

**Published:** 2019-09-19

**Authors:** Alba Miranda-Ribera, Maria Ennamorati, Gloria Serena, Murat Cetinbas, Jinggang Lan, Ruslan I. Sadreyev, Nitya Jain, Alessio Fasano, Maria Fiorentino

**Affiliations:** ^1^Department of Pediatric Gastroenterology and Nutrition, Mucosal Immunology and Biology Research Center, Massachusetts General Hospital, Boston, MA, United States; ^2^Department of Pediatrics, Harvard Medical School, Harvard University, Boston, MA, United States; ^3^Department of Molecular Biology, Massachusetts General Hospital, Boston, MA, United States; ^4^Department of Genetics, Harvard Medical School, Boston, MA, United States; ^5^Department of Pathology, Massachusetts General Hospital and Harvard Medical School, Boston, MA, United States

**Keywords:** gut permeability, microbial products trafficking, tight-junctions, zonulin transgenic mouse, microbiota, dysbiosis, immunity, chronic inflammatory diseases

## Abstract

The balanced interplay between epithelial barrier, immune system, and microbiota maintains gut homeostasis, while disruption of this interplay may lead to inflammation. Paracellular permeability is governed by intercellular tight-junctions (TJs). Zonulin is, to date, the only known physiological regulator of intestinal TJs. We used a zonulin transgenic mouse (Ztm) model characterized by increased small intestinal permeability to elucidate the role of a primary impaired gut barrier on microbiome composition and/or immune profile. Ztm exhibit an altered gene expression profile of TJs in the gut compared to wild-type mice (WT): Claudin-15, Claudin-5, Jam-3, and Myosin-1C are decreased in the male duodenum whereas Claudin-15, Claudin-7, and ZO-2 are reduced in the female colon. These results are compatible with loss of gut barrier function and are paralleled by an altered microbiota composition with reduced abundance of the genus *Akkermansia*, known to have positive effects on gut barrier integrity and strengthening, and an increased abundance of the *Rikenella* genus, associated to low-grade inflammatory conditions. Immune profile analysis shows a subtly skewed distribution of immune cell subsets toward a pro-inflammatory phenotype with more IL-17 producing adaptive and innate-like T cells in Ztm. Interestingly, microbiota “normalization” involving the transfer of WT microbiota into Ztm, did not rescue the altered immune profile. Our data suggest that a primary impaired gut barrier causing an uncontrolled trafficking of microbial products leads to a latent pro-inflammatory status, with a skewed microbiota composition and immune profile that, in the presence of an environmental trigger, as we have previously described (1), might promote the onset of overt inflammation and an increased risk of chronic disease.

## Introduction

The interplay among host genetics, immune response, intestinal microbiota composition and function, and exposure to environmental factors is critical in the breakdown of the normally coordinated, homeostatic mucosal immune tolerance leading to the development of chronic inflammatory disorders. In this scenario, the role of increased gut permeability has been recently appreciated and seems to be a critical factor in the chain of events leading to break of tolerance and onset of inflammation, whose sequence is still largely unknown. Increased intestinal permeability has been reported more than 30 years ago in celiac and Crohn's disease patients and first-degree relatives suggesting that barrier dysfunction may be necessary but not sufficient to disease development (2–5). Animal studies suggest that epithelial barrier dysfunction can trigger intestinal disease (6–9) as the development of spontaneous colitis in the IL-10 KO mouse is prevented by blocking increased small intestinal permeability with the zonulin inhibitor AT1001 (8). A recent *in-vitro* study using a human “gut-on-a-chip” model of inflammation that allows manipulations of the gut barrier and inflammatory factors, has shown that barrier dysfunction plays a critical role in the initiation of inflammation (10). Increased intestinal permeability has been proposed in the pathogenesis of several diseases not only affecting the gastrointestinal system but also metabolic disorders such as obesity and diabetes and/or diseases of the brain and the nervous system such as Parkinson's disease, multiple sclerosis, and the autism spectrum (11–16).

Intestinal hyperpermeability may precede the clinical manifestations of disease (2, 6, 17) but since inflammation can induce permeability defects (18–20), the controversy regarding the role of barrier defects as the cause or the consequence of disease has not been resolved. Growing evidence suggest mutual influence of impaired gut barrier, microbiome composition/function, and immune profile (21–23), with subsequent break of tolerance leading to chronic inflammatory diseases in genetically susceptible individuals. The currently accepted view is that besides genetic predisposition, exposure to environmental stimuli, loss of gut barrier function, inappropriate immune response, and changes in gut microbiota composition and function seem to be all at play in the pathogenesis of CID (24, 25). Also, intriguing is the evidence that these five factors can influence each other in an intricate “interactome” that remains largely undefined. Identifying the key elements that initiate break of intestinal mucosal tolerance is hence of paramount importance to guide the development of therapeutic approaches to specifically target the initiator of the entire cascade that leads to inflammation. Despite research efforts, a primary role of increased intestinal permeability in the onset of inflammation has not been established.

Pre-haptoglobin 2 (pHP2) is the archetype of the zonulin family (26) that reversibly regulates intestinal permeability by modulating intercellular TJs (27, 28) and its release is triggered by specific microbiota (29) and exposure to gliadin (30, 31). Zonulin is integrally involved in the pathogenesis of several CID (30–32) and it is augmented in chronic inflammatory conditions associated with TJ dysfunction, both at mucosal level (for example celiac disease) (28) and systemically (for example type-1 diabetes) (33). Zonulin plays a role as specific modulator of innate immune response involving antigen trafficking through both epithelial and endothelial compartments as well as M2 polarized macrophages expansion (22, 34).

Mice do not express pHP2, which is present only in humans (26). The Zonulin transgenic mouse (Ztm), genetically engineered to express murine pHP2 under its natural promoter (35), is a model of zonulin-induced increased intestinal permeability and represents a unique and extremely valuable tool to understand how loss of control of antigen trafficking may influence the gut microbiota composition and the immune system development.

In this study, we have leveraged on the Ztm as an animal model of increased gut permeability and inflammation susceptibility (1) to get insights on the role of the gut barrier in orchestrating early mucosal events that eventually lead to the onset of inflammation and development of disease.

## Materials and Methods

### Animals

A colony of C57Bl/6 (WT) was maintained in our facility at Massachusetts General Hospital (MGH). Breeding pairs of Ztm were created as previously described (35), and generously donated by Andrew Levy. Both colonies were maintained in our facilities under standard conditions (12 h light: 12 h dark cycle, standard humidity, and temperature), and housed in separate cages within the same facility during the study. Animals were fed standard pellet food and water *ad libitum*. Mice 4–8 weeks of age were used in this study. All animal studies in this paper were approved by the Institutional Animal Care and Use Committee at the MGH (2013N000013).

### Total RNA Extraction and Quantitative PCR (Real-Time PCR)

WT and Ztm mice were anesthetized using Forane (isoflurane, USP) from Baxter, and euthanized by cervical dislocation. Tissues (duodenum, jejunum and colon) were immediately collected and kept at −80°C until processed. Total RNA was extracted from frozen tissues using TRIzol™ reagent (Thermo Fisher) and the Direct-Zol RNA mini prep Kit (ZymoReserach) following the manufacturer's protocol. To ensure full removal of genomic DNA, samples were treated with DNA free™ Kit (Thermo Fisher,). Total mRNA was quantified and A260/280 and A260/230 measured using a Nanodrop 2000 (Thermo Fisher). cDNA was made with random hexamer primers plus Maxima H^−^ First Strand cDNA Synthesis Kit (Thermo Fisher) according to manufacturer's instructions. Quantitative real-time PCR was performed using PerfeCTa SYBR® Green SuperMix (Quanta) and run in a Bio Rad CFX connect thermocycler. All primers were designed in house (Table 1) using NIH's Primer Blast and obtained from Integrated DNA Technologies. We analyzed for gene expression the two most proximal sections of the small intestine (duodenum and jejunum). Only the sections of the intestine where we found significant gene expression changes are shown on graphs.

**Table 1:** List of all primers and FASTA accession numbers of the genes analyzed by real time qPCR analysis.

**Gene**	**Accession number**	**5^**′**^ OLIGO**	**3^**′**^ OLIGO**	**Gene function**
Claudin 1	NM_016674.4	GGCTTCTCTGGGATGGATCG	CTTTGCGAAACGCAGGACAT	Barrier-forming
Claudin 2	NM_016675.4	CCGTGTTCTGCCAGGATTCTC	AGGAACCAGCGGCGAGTAG	Pore-forming
Claudin 3	NM_009902.4	CCTAGGAACTGTCCAAGCCG	CCCGTTTCATGGTTTGCCTG	Barrier-forming
Claudin 4	NM_009903.2	CGTAGCAACGACAAGCCCTA	TGTCCCCAGCAAGCAGTTAG	Barrier-forming
Claudin 5	NM_013805.4	GTTAAGGCACGGGTAGCACT	TACTTCTGTGACACCGGCAC	Barrier-forming
Claudin 7	NM_016887.6	GCATACTTTCTGGGGGCCA	TGAAGCGACACTCTCACAGC	Barrier-forming claudin
Claudin 8	NM_018778.3	AAGGTCTACGACTCCCTGCT	TTCACGTTCTCATCGTCCCC	Barrier-forming claudin
Claudin 10	NM_001160096.1	CCCAGAATGGGCTACACATA	CCTTCTCCGCCTTGATACTT	Pore-forming claudin
Claudin 12	NM_001193661.1	GAGCCGATGTGCTCCTGTT	GGAGGGCTTGAGCTGTATGG	Barrier-forming
Claudin 15	NM_021719.4	AGGCACACCTTATCTGGCAC	TGCCCCCTGAACAATCACAA	Pore-forming claudin
INFγ	NM_008337.4	CAGCAACAGCAAGGCGAAA	CTGGACCTGTGGGTTGTTGAC	Pro-inflammatory cytokine
IL6	NM_031168.2	GTCCTTCCTACCCCAATTTCCA	CGCACTAGGTTTGCCGAGTA	Pro-inflammatory cytokine
IL8	NM_008176.3	ACTCAAGAATGGTCGCGAGG	GTGCCATCAGAGCAGTCTGT	Pro-inflammatory cytokine
IL10	NM_010548.2	TGGGTTGCCAAGCCTTATCG	TTCAGCTTCTCACCCAGGGA	Anti-inflammatory cytokine
IL17	NM_010552.3	TTTAACTCCCTTGGCGCAAAA	CTTTCCCTCCGCATTGACAC	Pro-inflammatory cytokine
JAM3	NM_023277.4	GCTGTGAGGTCGTTGCTCTA	AGTGGCACATCATTGCGGTA	Barrier-forming
Myo1C	NM_008659.3	CCGATCACCCGAAGAACCAA	CGCCGGAGGTTCTCAATGAA	Scaffolding
Occldn	XM_011244634.2	CTGACTATGCGGAAAGAGTTGAC	CCAGAGGTGTTGACTTATAGAAAGAC	Barrier-forming
TNFα	NM_013693.3	GATCGGTCCCCAAAGGGATG	TTTGCTACGACGTGGGCTAC	Pro-inflammatory cytokine
TRIC	XM_006517605.1	TGTGTGAAGCTGCCATCAGT	TTTGCCACGTAGTCAGGCAT	Barrier-forming
Zonulin	Sturgeon et al. (1)	GAATGTGAGGCAGATGACAG	GTGTTCACCCATTGCTTCTC	TJ modulator
ZO1	NM_009386.2	AAGAAAAAGAATGCACAGAGTTGTT	GAAATCGTGCTGATGTGCCA	Scaffolding
ZO2	NM_001198985.1	AGCTTGTAGTTCTGAGCCGC	CCGACACGGCAATTCCAAAT	Scaffolding
ZO3	NM_001282096.1	GGCTGATTGTTTCCAGGCCC	CCAGAGACAGCTATGCCGAA	Scaffolding
18S	X03205	AGAAACGGCTACCACATCCA	CCCTCCAATGGATCCTCGTT	Ribosomal RNA

*All primers were designed in house using NIH's Primer Blast and acquired from Integrated DNA Technologies (USA)*.

### Microbiota Analysis

#### Sample Collection and DNA Extraction

Stools were collected from a total of 71 mice divided into 3 groups: WT (*n* = 38), Ztm (*n* = 28), and Ztm_bWT_ (*n* = 5) (Ztm receiving bedding from WT). To minimize cage effects (36), mice within each genotype were cohoused for 2 weeks before sample collection. Fecal samples were collected from each animal, flash frozen, and kept at −80°C until further processing. Genomic DNA, for each sample, was extracted using the DNeasy powersoil extraction kit (Qiagen) following Qiagen's instructions. DNA was quantified using Nanodrop. In order to carry out the phylogenetic profiling, the hypervariable V4 region of the 16S rRNA gene was amplified by PCR using 5X prime master mix (Prime), reverse 806 primers were barcoded and a unique forward 515 primer (Integrated DNA Technologies) was used. To confirm correct amplification of the V4 regions, a regular gel electrophoresis was run. PCR products were purified using the QIAquick PCR purification kit (Qiagen) and their concentration was measured by Quant-iT Picogreen dsDNA kit following manufacturer's instructions. Sequencing of the samples was done at the MGH NextGen Sequencing Core facility (Boston, US), on the Illumina system using the MiSeq v2 500 cycles reagent kit as per manufacturer's instructions. To allow maximum coverage of the amplicon the system sequenced a total of 250 paired-end cycles. The following primers were used for the sequencing (37):

read 1 (TATGGTAATT GT GTGYCAGCMGCCGCGGTAA)

read 2 (AGTCAGCCAGCCGGACTACNVGGGTWTCTAAT)

index (AATGATACGGCGACCACCGAGATCTACACGCT).

### Bedding Transfer

Timed mating of C57BL/6 (WT) and Ztm mice were set up and monitored for live births. On the day of birth, half the soiled bedding from a WT cage was mixed with autoclaved sterile bedding in a new cage. Ztm breeders with their newborn pups were transferred to this new cage with WT bedding (Ztm_bWT_ mice). This process was repeated twice a week for 4 weeks until the pups were weaned. Colonic fecal content was collected 28 days after birth and analyzed for microbial composition.

### Preparation of Lymphocytes and Flow Cytometry

Spleens were mechanically disrupted, and single cell suspensions were depleted of RBCs with ACK lysis buffer (Invitrogen). Lymphocytes from intestines were prepared as described (38). Small and large intestines from 28 to 35 days old mice were collected in ice-cold HBSS, cleaned and chopped into 1 mm pieces. Samples were pooled from 2 mice and intestinal epithelial cells were removed by shaking in HBSS containing 5 mM EDTA, 1 mM DTT, 5% FCS, and 15 mM HEPES for 30 min at 37°C. Tissues were digested in enzyme solution prepared in EDTA-free HBSS buffer containing 0.15 mg/mL Liberase TL (Roche) and 0.1 mg/mL DNase1 (Roche) for 45 min at 37°C. The LPLs liberated into the supernatant were filtered in a 40 μm strainer and stained for flow cytometry.

Cells were first stained for surface markers and then fixed in eBioscience Fix/Perm buffer. Cells were subsequently permeabilized in eBioscience permeabilization buffer and stained with antibodies against transcription factors. All data were acquired on custom-built BD FACS Aria or Fortessa X20 and data analyzed using FlowJo software (Treestar, CA). mCD1d-PBS57 tetramers were from NIH tetramer core. All batches of reagent were titrated for optimal staining of C57BL/6 splenocytes, with unloaded CD1d tetramers serving as negative controls. Changes in antibody staining panels (e.g., antibody clones, reagent batches) across experiments were minimized. Strong discrepancies in staining from different experiments were noted and the data in question were excluded from final analyses.

### Microbiota Data Analysis

Sequencing data were processed and analyzed with QIIME2 software package v. 2018.2.0 (39). The sequencing reads with low quality score (average Q < 25) were truncated to 240 bp followed by filtering using *deblur* algorithm with default settings (40). The remaining high quality reads were aligned to the reference library using *mafft* (41). Next, the aligned reads were masked to remove highly variable positions, and a phylogenetic tree was generated from the masked alignment using the FastTree method (42). Alpha and beta diversity metrics and Principal Component Analysis plots based on Jaccard distance were generated using default QIIME2 plugins (39). Taxonomy assignment was performed using *feature-classifier* method and naïve Bayes classifier trained on the Greengenes 13_8 99% operational taxonomic units (OTUs). Differential abundance analysis of OTUs was performed using ANCOM (43). The Kruskal-Wallis test was used to assess the statistical significance of abundance differences, with the multiple testing corrections using Benjamini-Hochberg false discovery rate (FDR). The FDR cutoff was set at 0.05.

### Statistical Analysis

**Figure 1:**
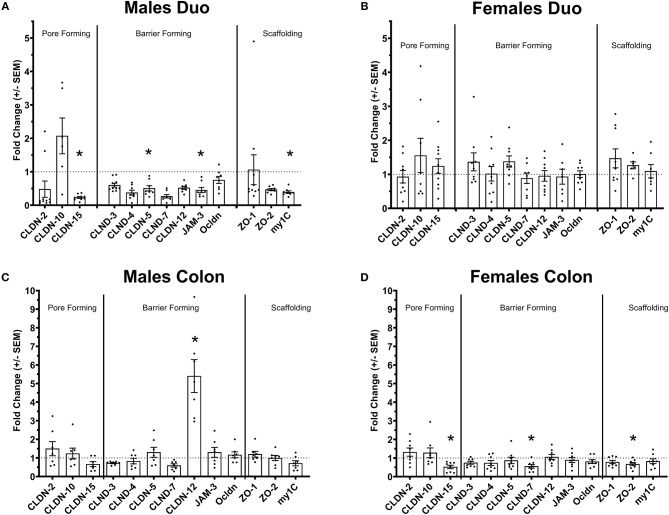
Tight-junctions gene expression profile in the intestine of Ztm. **(A)** Down regulated tight-junction genes in the duodenum Ztm males (*n* = 6–10). **(B)** Unaltered tight-junction profile in the duodenum of Ztm females (*n* = 7–12). **(C)** Increased *CLDN-12* in the colon of Ztm males (*n* = 7). **(D)** Downregulation of tight junction genes in the colon of Ztm females (*n* = 8). mRNA expression was analyzed by qPCR, normalized to WT, and expressed as fold regulation. Statistical analysis was performed by the non-parametric Mann-Whitney test comparing each gene to its WT counterpart. *p < 0.05.

**Figure 2:**
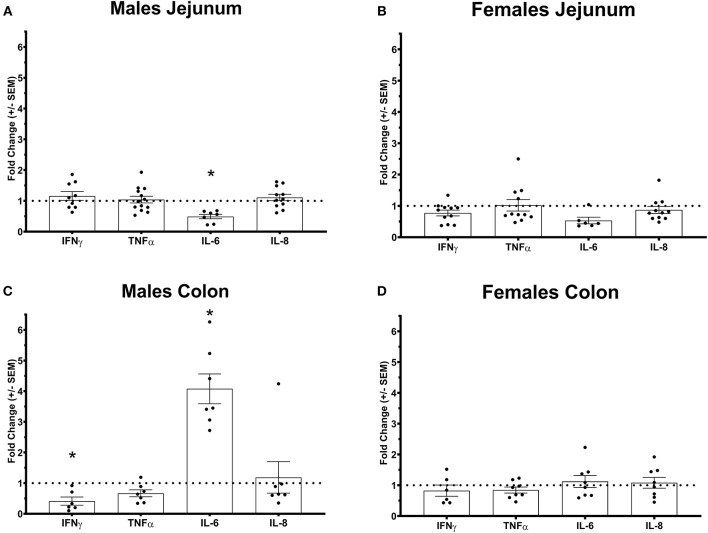
Pro-inflammatory cytokines gene expression profile in Ztm intestine: **A)** Down-regulated IL-6 in the jejunum of Ztm males (*n* = 13). **B)** Pro-inflammatory cytokines profile in and g the jejunum of Ztm females (*n* = 10). **C)** Down-regulated IFN up-regulated IL-6 in the colon Ztm males (*n* = 7). **D)** Unaltered Pro-inflammatory cytokines profile in the colon of Ztm females (*n* = 8). mRNA expression was analyzed by qPCR, normalized to WT, and expressed as fold regulation. Statistical analysis was performed by the non-parametric Mann-Whitney test comparing each gene to its WT counterpart. *p < 0.05.

Statistical analysis was performed in Graph Pad Prism 8, all data are expressed as means ± standard error of the mean (SEM) using the non-parametric Mann-Whitney test for comparisons between 2 groups for the qPCR analysis and unpaired parametric *t*-test for comparisons between 2 groups for the flow cytometry data. *P* < 0.05 was considered statistically significant in both tests. Figures 1, 2 represent the expression levels of the genes analyzed. Multiple genes are shown in each graph for convenience. Please note that each gene has been compared to its WT counterpart by the method described above and represented as fold change. In Table 2 is reported the fold change of all genes analyzed. Zonulin gene expression in the Ztm intestine has been expressed as dCt (Table 2) since no zonulin is present in the WT.

**Table 2:** **(A)** List of all genes analyzed by real time PCR in the duodenum, jejunum, and colon of adult mice (*n* = 6–12).

**A)**	**Duodenum**				**Jejunum**				**Colon**			
**Gene**	**Males**		**Females**		**Males**		**Females**		**Males**		**Females**	
	**FC**	***p***	**FC**	***p***	**FC**	***p***	**FC**	***p***	**FC**	***p***	**FC**	***p***
CLDN 1	0.02	ns	1.43	ns	–	–	–	–	2.19	ns	1.07	ns
CLDN 2	0.48	ns	0.93	ns	0.99	ns	1.22	ns	1.50	ns	1.32	ns
CLDN 3	0.60	ns	1.37	ns	1.56	ns	1.17	ns	0.72	ns	0.74	ns
CLDN 4	0.37	ns	1.02	ns	0.90	ns	1.13	ns	0.83	ns	0.73	ns
CLDN 5	0.51	**<0.05**	1.38	ns	1.22	ns	1.17	ns	1.30	ns	0.87	ns
CLDN 7	0.27	ns	0.89	ns	0.92	ns	1.13	ns	0.59	ns	0.55	**<0.05**
CLDN 8	–	–	–	–	–	–	–	–	1.83	ns	1.45	ns
CLDN 10	2.08	ns	1.55	ns	1.20	ns	0.94	ns	1.24	ns	1.28	ns
CLDN 12	0.52	ns	0.95	ns	1.00	ns	1.14	ns	5.40	**<0.01**	1.06	ns
CLDN 15	0.23	**<0.05**	1.86	ns	1.65	ns	0.89	ns	0.66	ns	0.52	**<0.05**
INFγ	bdl	–	bdl	–	1.89	ns	1.37	ns	0.41	**<0.05**	0.82	ns
IL6	bdl	–	1.27	ns	0.46	**<0.05**	0.54	ns	6.66	**<0.001**	1.13	ns
IL8	0.41	ns	2.50	ns	1.11	ns	0.87	ns	1.19	ns	1.08	ns
IL10	–	–	–	–	–	–	–	–	1.07	ns	1.04	ns
IL17	–	–	–	–	–	–	–	–	0.55	ns	bdl	-
JAM3	0.45	**<0.05**	0.93	ns	0.97	ns	0.99	ns	1.30	ns	0.89	ns
Myo1C	0.39	**<0.001**	1.09	ns	–	–	–	–	0.71	ns	0.82	ns
Occldn	0.75	ns	1.01	ns	–	–	–	–	1.17	ns	0.63	ns
TNFα	0.65	ns	2.69	ns	1.04	ns	1.02	ns	0.67	ns	0.85	ns
TRIC	0.53	ns	1.67	ns	–	–	–	–	1.76	ns	1.00	ns
ZO1	1.06	ns	1.47	ns	1.10	ns	0.76	ns	1.20	ns	0.78	ns
ZO2	0.47	ns	1.26	ns	0.85	ns	1.82	ns	0.99	ns	0.67	**<0.05**
ZO3	–	–	–	–	1.09	ns	0.99	ns	1.45	ns	1.16	ns
**B)**	**Duodenum**				**Jejunum**				**Colon**			
**Gene**	**Males**		**Females**		**Males**		**Females**		**Males**		**Females**	
	**dCt**	**st.dev**	**dCt**	**st.dev**	**dCt**	**st.dev**	**dCt**	**st.dev**	**dCt**	**st.dev**	**dCt**	**st.dev**
zonulin	17.2	1.25	16.3	0.70	14.2	2.37	15.5	1.13	14.5	1.02	15.8	1.75

*Expressed as Fold Change relative to same gene expression in the WT (FC, bdl = below detection level), statistics calculated with the non-parametric Mann-Whitney test (ns = non-significant, significant p-values provided in bold) comparing gene expression from the Ztm to its WT counterpart. **B)** Zonulin expression in the Ztm mice (males and females) duodenum, jejunum, and colon. Data are expressed as dCt (Ct normalized to its own 18S expression); standard deviation (st.dev) is also reported*.

## Results

### Ztm Mice Have an Altered TJ Gene Expression Profile in the Intestine

We have previously shown that Ztm mice exhibit a significantly increased small intestinal permeability both *in-vivo* and *in-vitro* at baseline associated to a higher susceptibility to DSS colitis, particularly in male mice, compared to WT (1). To elucidate the molecular basis of this altered permeability, we performed real time PCR of TJ and pro-inflammatory genes in different sections of the intestine (i.e., small intestine and colon) of WT and Ztm. Our results show an altered regulation of several TJs genes in Ztm compared to WT (Figure 1). In the small intestine of male Ztm we observed a significantly reduced expression of *Claudin* (*CLDN*)*-15, CLDN-5, JAM-3*, and *Myo1C* (*p* < 0.05; Figure 1A). We did not detect alterations in the expression of any of the analyzed genes in the small intestine of female Ztm (Figure 1B), in line with our previous finding of higher susceptibility to DSS-induced increased morbidity and mortality in male mice (1). Conversely, while in the colon of male Ztm only *CLDN-12* gene expression appeared significantly (*p* < 0.05) increased (Figure 1C), in the female colon *CLDN-15, CLDN-7*, and *ZO-2* were significantly (*p* < 0.05) reduced (Figure 1D). When looking at the expression of pro-inflammatory genes in the small intestine and colon, we detected a significant downregulation of IL-6 expression in the jejunum (*p* < 0.05) and its upregulation in the colon (*p* < 0.05) in Ztm males compared to WT males (Figures 2A,C). IFNγ was also significantly downregulated in the Ztm male colon (*p* < 0.05). Females exhibited a slightly decreased IL-6 expression in the jejunum (Figure 2B), while no changes were observed in the colon (Figure 2D) in any of the inflammatory cytokines analyzed. Please see Table 2 for the fold change of all genes analyzed.

### Ztm Shows Gut Dysbiosis Skewed Toward Inflammation

**Figure 3:**
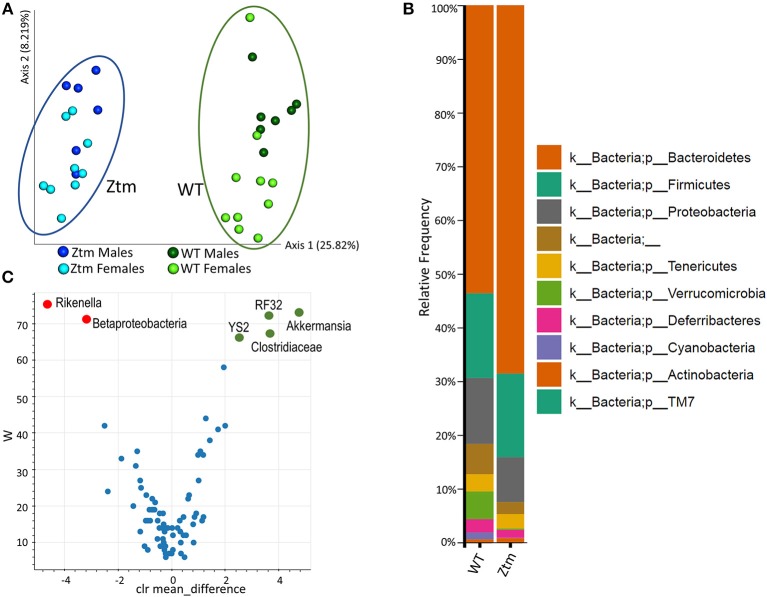
Baseline dysbiosis in Ztm gut microbiota. Stools from 15 WT mice (WT males *n* = 6, WT females *n* = 9) and 17 Ztm (Ztm males *n* = 7, Ztm females *n* = 10) were analyzed. **(A)** Principal Component Analysis (PCA) of Jaccard distances for Ztm (*n* = 17) and WT (*n* = 15) mice show distinct clustering between Ztm and WT. No significant differences between males and females within each group were detected. **(B)** Microbiota composition at phylum level. WT (*n* = 15), Ztm (*n* = 17). Males and females within each group were analyzed together. Absence of Verrucomicrobia and Cyanobacteria in the Ztm is evident. **(C)** Volcano plot showing the statistical differences between Ztm and WT at genus level. In green overrepresented genus in WT mice, in red overrepresented genus in Ztm. Differential abundance analysis of OTUs was performed using ANCOM. Statistical significance was tested by the Kruskal-Wallis of abundance differences, with the multiple testing corrections using Benjamini-Hochberg false discovery rate (FDR) set at 0.05.

To investigate whether constitutively increased gut permeability affects the host gut microbial composition, we analyzed the microbiome of 4–8 weeks old Ztm and WT mice. The gut microbiota composition in 4–8 week mice is established (44) but still susceptible to fluctuations by external factors (i.e., diet). Hence, this timeframe represents a window of opportunity to influence changes in the immune system and microbiome composition, and as such to investigate the impact of gut permeability. Principle component analysis (PCA) showed that Ztm microbiota clustered distinct from WT gut microbiota (Figure 3A). Analysis at phylum level showed that microbiota in Ztm was characterized by a marked reduced abundance of Verrucomicrobia (0.2%) compared to WT (5.2%), and the almost complete absence of Cyanobacteria (Ztm = 0.1% WT = 1.3%) (Figure 3B). At the class level, Clostridiacea were also significantly reduced in Ztm compared to WT. A deeper analysis confirmed a significantly reduced abundance of the orders RF32 (Alphaproteobacteria) and YS2 (Cyanobacteria) and the genus *Akkermansia* in Ztm compared to WT mice. Conversely, Betaproteobacteria and the genus *Rikenella* were significantly more abundant in the gut of Ztm compared to WT (Figure 3C), pointing to a pro-inflammatory microbiota composition in Ztm.

**Figure 4:**
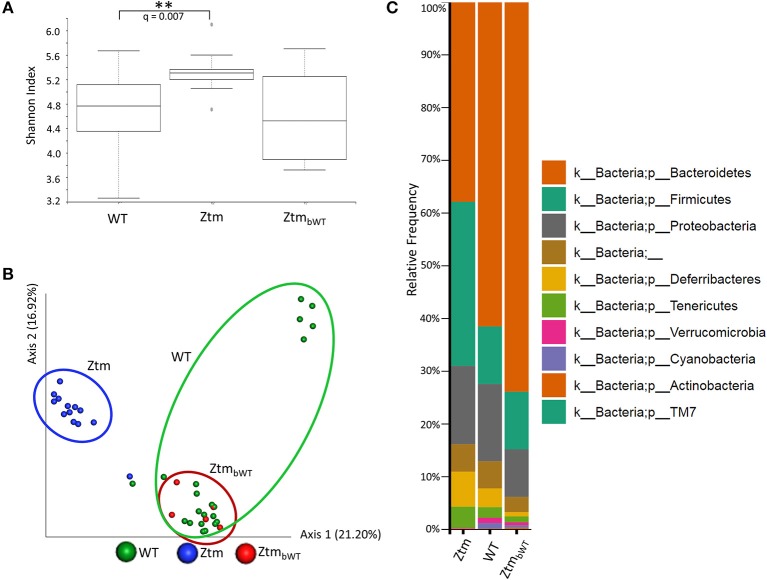
Microbiota analysis of Ztm mice with and without bedding transfer. Stools recovered from the colon of both male and female pups were analyzed as described in the material and methods. Stools were collected at weaning. Ztm_bWT_ represent pups that were subjected to bedding transfer from WT. Bedding transfer was carried out twice a week since birth. **(A)** Shannon Index, expression of alpha diversity (variation/complexity of the microbiome within the group). WT *n* = 21, Ztm *n* = 13, Ztm_bWT_ *n* = 5. WT and Ztm have a significantly different (*q* = 0.007) microbiota diversity, whereas no statistical difference is observed between WT and Ztm_bWT._ **(B)** Microbiota composition at phylum level. Successful engraftment of Verrucomicrobia and Cyanobacteria from WT to Ztm_bWT_ following bedding transfer. **(C)** Principal component Analysis (PCA) of Jaccard distances. Ztm_bWT_ (red) cluster with WT (green) and separately from Ztm (in blue). Differential abundance analysis of OTUs was performed using ANCOM. Statistical significance was tested by the Kruskal-Wallis of abundance differences, with the multiple testing corrections using Benjamini-Hochberg false discovery rate (FDR) set at 0.05. **q < 0.01.

To understand whether changes in the Ztm microbiota affected the development of the immune system (described below), we also analyzed the microbiota in Ztm mice that had received at birth bedding from a cage housing WT mouse (Ztm_bWT_, Ztm receiving bedding from WT) with the goal of normalizing microbiota between WT and Ztm mice. The Shannon Diversity Index (indication of the microbiota's complexity within the group) showed a significant difference between WT and Ztm (*q* = 0.007) but not between WT and the bedding-transferred Ztm_bWT_ (Figure 4A). Consistently with the Shannon Diversity Index, the PCA plot showed that Ztm microbial composition segregated from the WT cluster, while Ztm_bWT_ microbiota clustered with the WT microbiota (Figure 4B), indicating the successful engraftment of WT microbiota in the Ztm_bWT_. Verrucomicrobia and Cyanobacteria, that were severely reduced in Ztm, were engrafted in Ztm_bWT_ where we observed the successful colonization of *Akkermansia* (Figure 4C) and, normalization of *Rikenella* abundance (data not shown).

### Immune Phenotype of Ztm Mice

Changes in intestinal barrier function may cause bacterial products including endotoxins and other molecules to leak through the gut barrier and disseminate in both the lamina propria and systemically, potentially instigating changes in the immune phenotype. To determine if increased intestinal permeability and/or an altered microbiota influence lymphocyte distribution in the gut and in secondary lymphoid organs, we compared WT mice with Ztm and Ztm_bWT_, as described in the above paragraph (Figures 4B,C).

**Figure 5:**
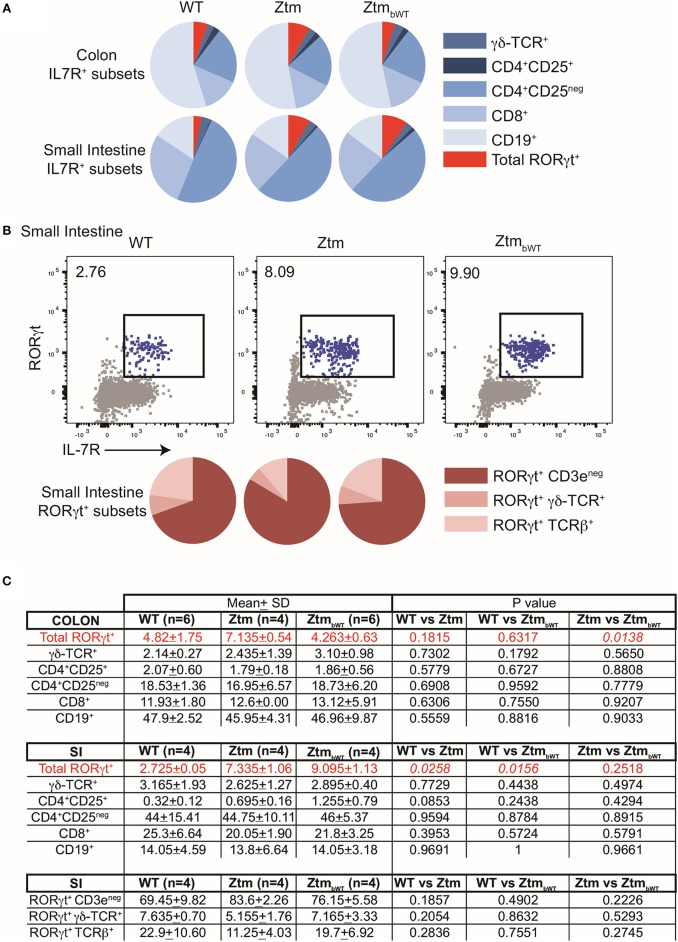
Distribution of immune cell subsets in colon and SI of WT and Ztm/Ztm_bWT_ mice. Lamina propria and epithelial compartment lymphocytes from the colon (WT *n* = 6; Ztm *n* = 4; Ztm_bWT_ *n* = 6) and small intestine (SI) (WT *n* = 4; Ztm *n* = 4; Ztm_bWT_ *n* = 4) of 35 days old mice were analyzed by flow cytometry. **(A)** Pie chart representation of distribution of indicated IL7R^+^ subsets in colon (Top row) and small intestine (SI) (Bottom Row). **(B)** (Top row) Representative flow cytometry dot plots show expression of IL-7R (x-axis) and RORγt (y-axis) in lymphocytes from the SI. (Bottom Row) Pie chart representation of distribution of indicated IL7R^+^RORγt^+^ subsets in SI. **(C)** Summary of distribution of immune cell subsets in colon and SI. Mean, SD, and *p*-values are indicated. Unpaired parametric *t*-test was used for comparisons between the 2 groups with significance set at *p* < 0.05.

**Figure 6:**
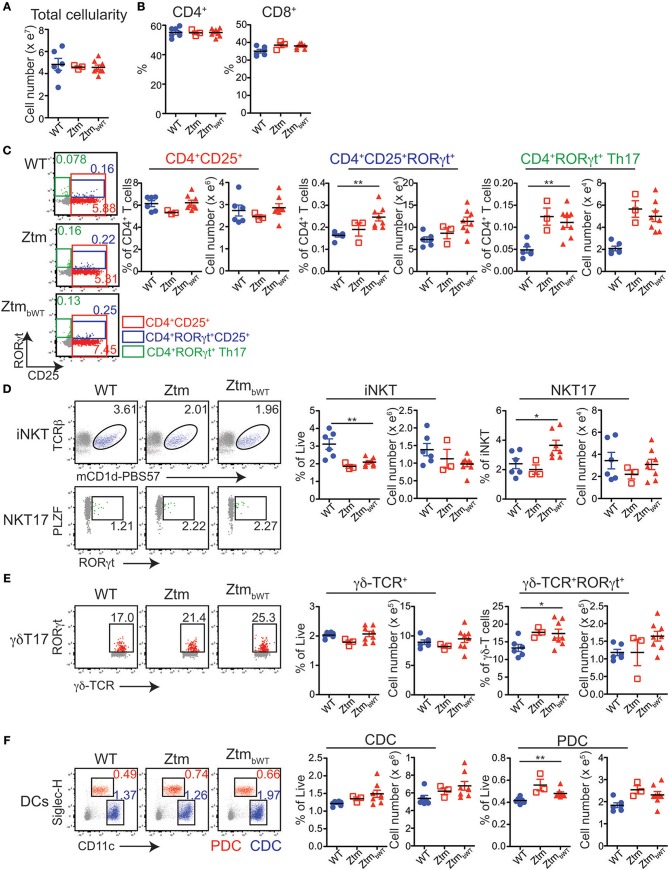
Distribution of immune cell subsets in spleen of WT and Ztm/ZtmbWT mice: single cell preparations from the spleen of 28–35 days old WT (*n* = 6), Ztm (*n* = 3), and ZtmbWT (*n* = 8) mice analyzed by flow cytometry. **(A)** Total splenic cellularity. **(B)** Vertical scatter plots show frequency of IL7R+TCRβ+ CD4+ and CD8+ T cells. **(C)** (Left) Representative flow cytometry dot plots show expression of CD25 (x-axis) and RORγt (y-axis) on CD4+ T cells. (Right) Vertical scatter plots show frequency and cell numbers of CD4+CD25+ cells, CD4+CD25+RORγt+ cells, and CD4+RORγt+ Th17 cells. **(D)** (Left) Representative flow cytometry dot plots show expression of (Top row) mCD1d-PBS57 tetramer (x-axis) and TCRβ (y-axis) on IL7R+ cells and (Bottom row) RORγt (x-axis) and PLZF (y-axis) on mCD1d-PBS57 tetramer+ TCRβ+ iNKT cells. (Right) Vertical scatter plots show frequency and cell numbers of iNKT cells and RORγt+ NKT17 cells. **(E)** (Left) Representative flow cytometry dot plots show expression of δ-TCR (x-axis) and RORγt (y-axis) on IL7R+ cells. (Right) Vertical scatter plots show frequency and cell numbers of γδ T cells and RORγt+ γδ-T17 cells. **(F)** (Left) Representative flow cytometry dot plots show expression of CD11c (x-axis) and Siglec-H (y-axis) on live cells (Red cells: CD11cloSiglec-H+ plasmacytoid dendritic cells or PDCs; Blue cells: CD11chiSiglec-Hneg conventional dendritic cells or CDCs). (Right) Vertical scatter plots show frequency and cell numbers of CDCs and PDCs. Data in **(A–F)** are representative of 3 independent experiments. Error bars are SEMs. Unpaired parametric *t*-test was used for comparisons between 2 groups with significance set at ^*^p < 0.05 and ^**^p < 0.01.

Overall, Ztm and Ztm_bWT_ mice had similar distribution of major immune cell subsets compared to WT mice (Figures 5, 6). Flow cytometry analysis of lymphocytes in the colon and small intestine revealed no significant differences in the frequency of IL7R^+^ conventional γδ-TCR^+^ cells, CD4^+^CD25^+^ cells, conventional CD4^+^CD25^neg^ and CD8^+^ T cells, and CD19^+^ B cells (Figures 5A,C). However, we noted an overall statistically significant increase in the frequency of IL7R^+^RORγt^+^ cells in the small intestine but not in the colon of both Ztm and Ztm_bWT_ mice (Figure 5A, red slice; Figures 5B,C). This increase was most likely due to elevated IL7R^+^CD3^neg^RORγt^+^ cells that may be innate lymphoid cells (ILCs) (Figure 5B, bottom row; Figure 5C). Splenic cellularity was also comparable across all groups (Figure 6A) as was the distribution of CD4^+^ and CD8^+^ T cells (Figure 6B). There was no significant difference in the frequency of CD4^+^CD25^+^ cells in the spleen of Ztm and Ztm_bWT_ mice compared to WT mice (Figure 6C). However, the frequency and numbers of CD4^+^CD25^+^ cells expressing RORγt^+^ was significantly increased in Ztm_bWT_ mice (Figure 6C). We also noted an increased frequency and numbers of CD4^+^RORγt^+^ Th17 cells in both Ztm and Ztm_bWT_ mice (Figure 6C). Further analyses revealed an interesting distribution of transcription factor RORγt-expressing innate-like T cells. While the frequency and numbers of mCD1d-PBS57 tetramer^+^ invariant NKT (iNKT) cells was decreased, the frequency of RORγt expressing subset of iNKT cells (NKT17 cells) increased in both Ztm and Ztm_bWT_ compared to WT mice (Figure 6D). Similarly, there was a modest increase in the frequency of γδ T cells (γδ TCR^+^ innate-like T cells) that expressed RORγt (Figure 6E). NKT17 cells and γδ-17 T cells are pro-inflammatory innate-like T cell subsets that produce IL-17 and have been implicated in the pathogenesis of various autoimmune diseases including type 1 diabetes and celiac disease (45, 46). Finally, we determined the frequency and phenotype of conventional CD11c^hi^ dendritic cells (CDC) and CD11c^lo^SiglecH^+^ plasmacytoid dendritic cells (PDC) and noted a small but significant increase in the frequency of splenic PDCs but not CDCs in both Ztm and Ztm_bWT_ compared to WT mice (Figure 6F). These data suggest that altered gut permeability subtly increased the baseline frequency of IL-17 producing T cells in mucosal tissue and in secondary lymphoid organs of Ztm mice in the absence of overt inflammation and disease. The fact that the engraftment of WT microbiota did not affect the immune phenotype in Ztm_bWT_ (no significant differences were detected between Ztm and Ztm_bWT_), suggests that the increased trafficking of microbial products through an impaired gut barrier rather than the function of an imbalanced microbiota primarily imprints the development of the immune system in the Ztm.

## Discussion

We have previously shown that Ztm express zonulin, exhibit constitutive increased gut permeability *in-vivo* and are more susceptible to DSS induced colitis (1). The increased morbidity and mortality affected predominantly Ztm males and is supported by our molecular data showing an altered TJ gene expression profile in the duodenum of male Ztm, with the significant decreased expression levels of several genes suggesting an impairment of small intestinal barrier function. Although, its function of gut barrier- or pore-forming claudin still has not been well-characterized, *CLDN-12* significant increase in the colon of Ztm males could represent a compensatory mechanism to re-establish barrier function and/or electrolytes absorption (47). Of interest was the significantly reduced expression of *Myosin-1C* gene, because of its proven involvement in the zonulin pathway and its disassociation from ZO-1, leading to TJ disassembly and increased intestinal permeability (48). It's important to point out that although changes in gene expression not necessarily correspond to changes in protein expression, the fact that multiple TJ genes are affected, strongly suggests an impaired gut barrier. Also, intriguing are our findings of *IL6* gene expression upregulation in the colon of Ztm males (and not females), suggesting that the increased permeability and trafficking of microbial products in the duodenum generates a pre-inflammatory imprinting in the immune system predisposing Ztm to develop colitis when exposed to an external inflammatory stimulus (1). Increased colonic expression of *IL-6* might also be the consequence of a constitutive increased passage of microbiota species into the colonic lamina propria.

Gut permeability defects in the Ztm seem to have a critical effect on both the gut microbiota composition and the immune system. The analysis of stool microbiota showed dysbiosis in the Ztm gut. Striking was the marked reduced presence of *Akkermansia* (sp. *muciniphila*) vs. a significantly more abundant *Rikenella* genus in the Ztm gut. *A. muciniphila* contribute to enterocyte monolayer integrity and the overall strengthening of the gut epithelial barrier (49–51) and low levels have been associated to many human diseases (52–54) and have been reported as intestinal microbiota feature of both genetic and diet-induced obese and diabetic mice (55, 56).

While *A. muciniphila* are indicators of a gut healthy status, on the other end high abundance of *Rikenella* has been found in obesity and diabetes (57, 58) and hence associated with a status of low chronic non-infective inflammation. Overall, the low abundance of *A. muciniphila* and the enriched *Rikenella* presence in the gut of Ztm, suggest that the Ztm gut microbiota is skewed vs. a more maladaptive and pathogenic profile.

It is well-known that commensal bacteria are involved in the development of the immune system and the establishment of oral tolerance (59–61). The balance between pro-inflammatory IL-17^+^ helper Th17 cells and the anti-inflammatory Foxp3^+^ regulatory T cells (Treg) in the mucosa (62, 63) plays a key role in the development of chronic inflammatory diseases (64, 65). Microbial products have been shown to regulate the balance between Treg cells and IL-17 producing RORγt+ Th17 cells (59, 66). It has been shown that specific components of the commensal microbiota induce Th17 cells in the lamina propria of the small intestine and that antibiotic treatment prevented such differentiation (67).

In the Ztm, despite an altered baseline trafficking of microbial products, major immune cell subset distribution appears unaffected. However, the modest increase in pro-inflammatory IL-17 producing innate and innate-like cells in the intestine as well as in gut distal secondary lymphoid sites indicate that the threshold of immune reactivity in these mice might be altered, rendering them more susceptible to lose tolerance to non-self-antigens with subsequent onset of inflammation. In fact, when challenged with DSS, Ztm develop a more severe colitis and exhibit a significantly increased morbidity and mortality that is associated to elevated zonulin gene expression (1).

Immune profile studies following bedding transfer with the successful colonization of WT microbiota into Ztm mice, show no changes indicating that the Ztm immunophenotype is largely unaffected by the microbial composition and suggest that increased intestinal trafficking of microbial products primarily drives changes in the host immune system. In other words, the reduced barrier function in Ztm supersedes an imbalanced microbiota for the enhanced differentiation of RORγt^+^ and IL-17 producing subsets.

Our data indicate that zonulin-dependent increased gut permeability and microbial products trafficking orchestrate the development of the immune system and microbiota composition toward a pro-inflammatory status in the Ztm, with increased IL-17 producing cells and the lack of gut protective microbial species (i.e., *Akkermansia*) vs. an over-representation of pro-inflammatory strains (i.e., *Rikenella*). This low-grade pro-inflammatory-skewed status renders Ztm critically susceptible to develop overt inflammation leading to disease in the presence of exogenous stimuli (i.e., DSS) (1). Taken together, these data suggest that increased trafficking of microbial products in the small intestine might cause break of tolerance and onset of inflammation either locally or at distance. Indeed, the DSS experiments in Ztm (1) support our hypothesis of a key role of small intestinal microbial products trafficking in triggering inflammation at a distal site (colon). The observation that the onset of colitis with loss of the stem cell niche and its regenerative capacity can be rescued by blocking the zonulin pathway suggests a pivotal role of the mutually-influenced increased small intestinal trafficking of microbial products, a pro-inflammatory immune system, and gut dysbiosis shown in this paper, rather than merely a local effect of DSS once it reaches the colon. This is also in line with data generated in the IL-10 KO mouse, another model of colitis in which increased small intestinal permeability was shown to have a pathogenetic role in leading to colitis (8).

Although, further investigations are needed, these observations support our hypothesis and a new paradigm in which the presence in Ztm of an increased gut permeability, an altered immune system and dysbiosis of the gut microbiota are not sufficient to initiate the chain of events leading to overt inflammation. In the absence of an exogenous stimulus, Ztm remain healthy. It is the action of an external trigger to drive Ztm over the threshold where they develop disease. The same chain of events can be hypothesized in CID, in which in genetically predisposed individuals, exogenous factors that trigger epithelial barrier dysfunction and activation of the immune system could selectively promote the growth of microbial species in the gut that in turn worsen changes in the intestinal tissue resulting in breaking mucosal tolerance and onset of chronic inflammation, locally, and/or systemically and clinical symptoms.

In conclusion, this study provides important insights on the understanding of the role of gut permeability in the initiation of inflammatory diseases, offering the basis for innovative and yet unexplored therapeutic approaches for the management of these debilitating chronic diseases aimed at re-establishing the intestinal barrier function by downregulating the zonulin pathway.

## Data Availability

All datasets generated for this study are included in the manuscript/supplementary files.

## Ethics Statement

This study was carried out in accordance with the recommendations and guidelines of the Institutional Animal Care and Use Committee at the MGH. The protocol was approved by the Institutional Animal Care and Use Committee at the MGH (2013N000013).

## Author Contributions

MF, AM-R, NJ, RS, and AF conceived and designed the experiments. AM-R, ME, GS, JL, and MC carried out the experiments. MF, AM-R, NJ, RS, MC, and AF contributed to the interpretation of the results. AM-R, MF, NJ, and AF wrote the manuscript. All authors provided critical feedback and helped shape the research, analysis, and manuscript.

### Conflict of Interest Statement

AF is a stock holder at Alba Therapeutics, serves as a consultant for Inova Diagnostics and Innovate Biopharmaceuticals, is an advisory board member for Axial Biotherapeutics and Ubiome, and has a speaker agreement with Mead Johnson Nutrition. The remaining authors declare that the research was conducted in the absence of any commercial or financial relationships that could be construed as a potential conflict of interest.
